# Supplementary material for: Cohort profile: Health and Attainment of Pupils in a Primary Education National Cohort (HAPPEN) – a hybrid total population cohort in Wales, UK
Source: BMJ Open. 2024 Dec 20;14(12):e088721. doi: 10.1136/bmjopen-2024-088721 (PMC11667454; doi:10.1136/bmjopen-2024-088721)
Supplement: Supplementary file 1 [file bmjopen-2024-088721.reviewer_comments.pdf]

PEER REVIEW HISTORY

BMJ Open publishes all reviews undertaken for accepted manuscripts. Reviewers are asked to complete a checklist review form and are provided with free text boxes to elaborate on their assessment. These free text comments are reproduced below.

ARTICLE DETAILS

Title (Provisional)

Cohort Profile: Health and Attainment of Pupils in a Primary Education National-Cohort (HAPPEN) – A hybrid total population cohort in Wales, UK

Authors

Locke, Amy; Kennedy, Jonathan L; Burdett, David W E; Jones, Hope; James, M; Marchant, Emily; Bandyopadhyay, Amrita; Stratton (GS), Gareth; Brophy, Sinead

---

VERSION 1 - REVIEW

**Reviewer**	**1**
**Name**	**Ram, Bina**
**Affiliation**	**Imperial College London - Charing Cross Campus, Department of Primary Care and Public Health**
**Date**	**17-Aug-2024**
**COI**	**None.**

Thank you for the opportunity to review this manuscript which describes the data of the health and wellbeing of children in Wales through the HAPPEN survey which informs the HAPPEN national cohort and its linkage with the SAIL databank. This is an important piece of research and the data provides opportunities for future research to monitor and examine the health and wellbeing of children in Wales.

Overall, this manuscript has been presented very well and informs the reader of the background of the HAPPEN survey and the population characteristics of those completing the survey. I have a few, minor points for the authors to consider/clarify in the introduction and discussion that may help improve the flow.

Introduction

1. Opening sentence of introduction, please add ‘years’ after ‘Children aged 7-11…’

2. Page 6, line 48: first mention of HAPPEN without an explanation of what HAPPEN is, but it is described in the second paragraph. I would therefore suggest removing the mention of HAPPEN here and consider amending the sentence to “The curriculum reform in Wales has a statutory focus on health and wellbeing. It is an autonomous, school level curriculum

through which interventions focused on individualised approaches to health and wellbeing are possible.”

3. The sentence removed from above can be placed in the next paragraph page 6 line 60 “….the introduction of the Curriculum for Wales (CfW) from September 2022 (9). This offers an array of opportunities for HAPPEN. The CfW places statutory…”

4. Page 7, line 33: the authors state that schools can participate in the HAPPEN survey throughout the year:

(a) Is the survey compulsory for all schools to complete?

(b) How do schools know about the survey?

(c) How do they register to take part and what is the process for them to receive the survey?

(d) What percentage of schools that have registered gone on to complete the survey?

5. Page 8, line 25: over 600 schools in the country have registered to take part – to provide context for the reader, it would be helpful to know what percentage this is of all schools in Wales.

Discussion

6. Wondered why the authors started with the strengths and limitations of the HAPPEN cohort. For better flow, I would suggest that the Discussion open with the ‘Research Aspirations’ and ‘Data availability and access’ sections. The strengths and limitations should then follow, and end with the ‘Future Aspiration’ section. However, if the authors can justify the current format, I will leave re-ordering to their discretion.

7. Can the authors comment on whether the demographics of the HAPPEN cohort are representative of the school population as a whole in Wales for those year groups?

Table 1

8. For completeness, please add footnotes for acronyms used in table (‘WMID’) and provide references for the 2011 and 2019 WMID.

**Reviewer**	**2**
**Name**	**Widyastari, Dyah Anantalia**
**Affiliation**	**Mahidol University**
**Date**	**26-Aug-2024**
**COI**	**None.**

Cohort Profile: Health and Attainment of Pupils in a Primary Education National-Cohort (HAPPEN) – A hybrid total population cohort in Wales, UK

Comments for authors:

1. Generally, this paper has the potential to contribute to the body of knowledge in longitudinal studies of children's health and their academic attainment. However, there is insufficient data and analysis on the children's outcomes that supposed to provide an evidence-based from behavioral observation over the years. The only outcome presented was the deprivation 2011 and 2019 and no further analysis was performed.

2. The authors should be applauded for their attempts in linking anonymous data to external database (SAIL databank) to provide a more meaningful interpretation to the findings, yet the existing analysis is limited to only gender, ethnicity and deprivation that insufficiently describe the overall cohort profiles.

3. While the HAPPEN survey is likely to collect many information related to physical activity, dietary intake, and sleep, there is no data presented in the current analysis.

4. It was not clear whether the authors are also the research team of HAPPEN survey, or the authors utilizing the public domain data which is considered as secondary data analysis.

5. The authors have considered Patient and public involvement (PPI) as an integral part of the research where members of the public actively participate in shaping the research project. The authors also stated that the survey has involved local authorities, charities and organizations relating to children's development, health and wellbeing to ensure a range of priorities are captured. Hence, it was not clear how PPI may differentiate the outcomes.

6. While the authors stated that the HAPPEN cohort has been used to explore the health and well-being of children during the COVID-19 pandemic, or pupils' perception on free school meals (p.14), the analysis seemed to be conducted by other researchers, and not by the current team of authors.

7. Inadequate discussion – the authors do not discuss their findings, only focused on the strength of HAPPEN survey but was not on the current analysis.

8. Inconclusive – the descriptive data provided is insufficient to portray the cohort profile and the health outcomes.

VERSION 1 - AUTHOR RESPONSE

Reviewer: 1

Introduction

1. Opening sentence of introduction, please add 'years' after 'Children aged 7-11...'

This has been amended. The sentence now reads 'Children aged 7-11 years typically enjoy good overall health.'

2. Page 6, line 48: first mention of HAPPEN without an explanation of what HAPPEN is, but it is described in the second paragraph. I would therefore suggest removing the mention of HAPPEN here and consider amending the sentence to "The curriculum reform in Wales has a statutory focus on health and wellbeing. It is an autonomous, school level curriculum through which interventions focused on individualised approaches to health and wellbeing are possible."

***Thank you for your suggestion. The mention of HAPPEN has now been removed from this sentence and 'The curriculum reform in Wales has a statutory focus on health and wellbeing. It is an autonomous, school level curriculum through which interventions focused on individualised approaches to health and wellbeing are possible'* has been added.**

3. The sentence removed from above can be placed in the next paragraph page 6 line 60 "….the introduction of the Curriculum for Wales (CfW) from September 2022 (9). This offers an array of opportunities for HAPPEN. The CfW places statutory…"

This has been amended and now reads 'This offers an array of opportunities for HAPPEN. The CfW places statutory focus on health and wellbeing as one of the six Areas of Learning and Experience (AoLE).'

4. Page 7, line 33: the authors state that schools can participate in the HAPPEN survey throughout the year:

(a) Is the survey compulsory for all schools to complete?

Thank you for highlighting an area in which we can provide further information regarding HAPPEN. We have added a few sentences to address this: "Schools are invited to take part in HAPPEN on a voluntary basis, with recruitment opening every September in line with the beginning of the new school term. Schools can choose to take part and have the autonomy to complete at a time that suits them, inside the academic year."

(b) How do schools know about the survey?

As above, we have added further information about this process: "Schools are notified that the survey can be completed via email recruitment in the first instance. Schools can register their interest and nominate a point of contact who will receive updates about the survey whereby they will learn more information about the survey. Wider recruitment takes

place through stakeholder engagement via local authority education teams, community development teams, charities and word of mouth."

(c) How do they register to take part and what is the process for them to receive the survey?

Again, thank you for highlighting an area which we can provide further information on. We have added more detail: "Schools register to take part in the HAPPEN Survey via the HAPPEN Website (www.happen-wales.co.uk). Once registered, the school will receive all the information they need to complete the survey including links to parent/child information sheets and a link to the survey which is completed online."

(d) What percentage of schools that have registered gone on to complete the survey?

More information has been added to answer this query: "Over the last four academic years, an average of 105 schools completed the survey equating to 15% of the registration list."

5. Page 8, line 25: over 600 schools in the country have registered to take part – to provide context for the reader, it would be helpful to know what percentage this is of all schools in Wales.

Further information has been added in answer to this query: "Over 600 schools in the country have registered to take part (which equates to 52% of the primary schools in Wales) and over 40,000 pupils have taken part between 2016-2023."

Discussion

6. Wondered why the authors started with the strengths and limitations of the HAPPEN cohort. For better flow, I would suggest that the Discussion open with the 'Research Aspirations' and 'Data availability and access' sections. The strengths and limitations should then follow, and end with the 'Future Aspiration' section. However, if the authors can justify the current format, I will leave re-ordering to their discretion.

Thank you for this comment. This restructuring has been amended as suggested

7. Can the authors comment on whether the demographics of the HAPPEN cohort are representative of the school population as a whole in Wales for those year groups?

In the Wellbeing of Wales (2022) Report from Welsh Government, it was stated that 90.6% of the Welsh population are White (Welsh, English, Scottish, NI or British with 2.9% of the population Asian (the second largest category). Our cohort is reflective of the majority. Similarly, our deprivation is representative with 20-23% in our cohort living in the most deprived quintile, with 29% in the Welsh report.

8. For completeness, please add footnotes for acronyms used in table ('WMID') and provide references for the 2011 and 2019 WMID.

This has been amended to include Welsh Index of Multiple Deprivation (WIMD)

References

WIMD 2011 GOV.WALES. (2011). Welsh Index of Multiple Deprivation (full Index update with ranks): 2011 | GOV.WALES. [online] Available at: https://www.gov.wales/welsh-index-multiple-deprivation-full-index-update-ranks-2011 [Accessed 18 Oct. 2024].

WIMD 2019 Welsh Government (2019). Welsh Index of Multiple Deprivation (full Index update with ranks): 2019. [online] GOV.WALES. Available at: https://www.gov.wales/welsh-index-multiple-deprivation-full-index-update-ranks-2019.

Reviewer: 2

1. Generally, this paper has the potential to contribute to the body of knowledge in longitudinal studies of children's health and their academic attainment. However, there is insufficient data and analysis on the children's outcomes that supposed to provide an evidence-based from behavioral observation over the years. The only outcome presented was the deprivation 2011 and 2019 and no further analysis was performed.

Thank you for your comment. To clarify, the goal of a cohort profile is to describe the cohort, its formation, and provide initial demographic details. It is not intended as a research article with extensive analyses and results. Future research papers using this cohort will present more data and analysis of the relevant outcomes.

2. The authors should be applauded for their attempts in linking anonymous data to external database (SAIL databank) to provide a more meaningful interpretation to the findings, yet the existing analysis is limited to only gender, ethnicity and deprivation that insufficiently describe the overall cohort profiles.

Thank you for this comment. As mentioned above, the purpose of this cohort profile is to describe the cohort not to present extensive analysis.

3. While the HAPPEN survey is likely to collect many information related to physical activity, dietary intake, and sleep, there is no data presented in the current analysis.

As mentioned above, the purpose of this cohort profile is to describe the cohort not to present extensive analysis. Future journal articles using the HAPPEN cohort will explore these factors

4. It was not clear whether the authors are also the research team of HAPPEN survey, or the authors utilizing the public domain data which is considered as secondary data analysis.

To clarify, the authors are members of the HAPPEN research team. We have added this to our contributorship statement: "Contributors: All authors provided substantial contributions to the piece of work. AL, HJ, MJ, EM and SB contributed to the conception of this article. AL, HJ, MJ, EM, GS and SB were involved in manuscript writing and revision. MJ EM, and SB were involved in data analysis and interpretation. AL is responsible for the overall content as the guarantor. All authors read and approved the final manuscript. All authors agree to be accountable for all aspects of the work. All co-authors are part of the HAPPEN research team whether they have been involved in the initial conception or ongoing management and analysis of findings."

5. The authors have considered Patient and public involvement (PPI) as an integral part of the research where members of the public actively participate in shaping the research project. The authors also stated that the survey has involved local authorities, charities and organizations relating to children's development, health and wellbeing to ensure a range of priorities are captured. Hence, it was not clear how PPI may differentiate the outcomes.

The Patient and Public Involvement section explains that HAPPEN was developed based on recommendations from head teachers. We emphasised the value of consultation, engagement, and collaboration throughout the process. PPI also contributed to shaping the survey questions. We have highlighted that while this collaboration doesn't influence the outcomes, it helps ensure the research addresses relevant questions.

6. While the authors stated that the HAPPEN cohort has been used to explore the health and well-being of children during the COVID-19 pandemic, or pupils' perception on free school meals (p.14), the analysis seemed to be conducted by other researchers, and not by the current team of authors.

Thank you for this comment. To clarify, there are various members of the HAPPEN team and the research presented has been conducted by team members.

7. Inadequate discussion – the authors do not discuss their findings, only focused on the strength of HAPPEN survey but was not on the current analysis.

This cohort profile focuses on describing the cohort rather than discussing research findings, as it is not a research article. The discussion outlines the cohort, the recruitment process, and the potential for future studies.

8. Inconclusive – the descriptive data provided is insufficient to portray the cohort profile and the health outcomes.

The descriptive data is intended to give an overview of the cohort's demographics. Health outcomes were not included in this data and will be examined in future articles using the cohort.

VERSION 2 - REVIEW

Reviewer 1
Name Ram, Bina
Affiliation Imperial College London - Charing Cross Campus, Department of Primary Care and Public Health
Date 15-Nov-2024
COI

Thank you to the authors for considering my comments. The additions have improved clarity.
